# A Comprehensive Beam-Level Assessment of Pencil-Beam Scanning Migration and Utilization at a Multi-System Proton Therapy Center

**DOI:** 10.1016/j.ijpt.2026.101969

**Published:** 2026-10-06

**Authors:** Perry B. Johnson, Twyla Willoughby, Jonathan Alvarez, Julien Forthomme, An Van den Bulck, Nancy P. Mendenhall

**Affiliations:** 1 University of Florida Health Proton Therapy Institute, Jacksonville, FL, USA; 2 University of Florida College of Medicine, Gainesville, FL, USA; 3 Ion Beam Applications SA, Louvain-la-Neuve, Belgium

**Keywords:** Proton therapy, Pencil-beam scanning, ProteusONE, ProteusPLUS, Proton operations

## Abstract

**Purpose:**

To provide a beam-level comparison of pencil-beam scanning (PBS) proton therapy at a high-volume center operating both the ProteusPLUS (P-PLUS) and ProteusONE (P-ONE) platforms and to evaluate how system capabilities influenced room utilization during a multi-year transition to PBS.

**Methods and Materials:**

Log files were analyzed for three rooms: P-PLUS Gantry Room 2 (GTR2; 2018-2024), P-PLUS Gantry Room 1 (GTR1; mid-2023-2024), and P-ONE Compact Gantry Room (CGTR; 2020-2024). A script was written to extract beam parameters while clinical and demographic information were obtained from the MOSAIQ database. Data streams were merged, then grouped and analyzed by site, age, and temporal factors.

**Results:**

The final dataset included 120,367 beams for the P-PLUS and 95,587 for the P-ONE. During an 18-month comparative period, mean throughput was 27.4 ± 5.4 patients/day (GTR2), 22.9 ± 5.1 (GTR1), and 27.0 ± 4.6 (CGTR), with mean session times of 28.2 ± 2.5, 25.6 ± 1.7, and 26.9 ± 2.4 minutes, respectively. Case allocation reflected platform/nozzle differences: GTR2 primarily treated prostate, breast, and thorax/abdomen targets, while GTR1 and CGTR focused on prostate, head-and-neck, brain, and CNS cases. CGTR treated more pediatric patients (25.2% vs 7.1% in GTR2 and 5.8% in GTR1) and cases involving anesthesia. Maximum cyclotron energy was used more on GTR2 compared to CGTR (7.5% vs 0.4%), while range-shifter use was similar (71.1% vs 67.0%); couch flips occurred in 77% of P-ONE treatments.

**Conclusions:**

Expanding PBS capacity substantially shifted room utilization and case mix, driven mainly by differences in spot and field size, and secondarily by imaging, layer-switching, and pediatric-oriented room features. The P-ONE’s smaller field size did not materially constrain prostate treatment with involved lymph nodes, frequent couch flips did not inhibit high operational capacity, and the P-ONE’s lower minimum energy reduced range-shifter reliance and the need for maximum cyclotron energy. This real-world analysis provides guidance for case selection, workflow optimization, and future proton system design.

## Introduction

Since 2006, the UF Health Proton Therapy Institute (UFHPTI) has operated a multi-room ProteusPLUS (P-PLUS) proton therapy (PT) system encompassing three gantry rooms and a fixed beamline [Ion Beam Applications SA (IBA), Louvain-la-Neuve, Belgium]. The system has treated over 10 000 patients and undergone several upgrades, converting 2 nozzles from passive scattering to pencil-beam scanning (PBS) and adding cone-beam CT (CBCT) to all gantry rooms. In 2019, the facility underwent a major renovation and build-out by adding 15 000 sqft to house a new ProteusONE (P-ONE) system (IBA, Louvain-la-Neuve, Belgium) and additional office space. Since then, over 2000 patients have been treated on the P-ONE, making UFHPTI the only facility worldwide operating both Proteus platforms to date.

The introduction of PBS through both system upgrades and facility expansion substantially changed clinical utilization patterns at UFHPTI, providing practical lessons for other centers undergoing similar transitions. Additionally, while the P-PLUS and P-ONE platforms share many features, they are distinct systems that offer different benefits and challenges, as well as ways to optimize their performance. UFHPTI is well-positioned to assess these areas based on its unique clinical experience, which affords direct comparison across a variety of facets, including technological features, performance metrics, treatment approaches, and operational characteristics. Such information is relevant for both current and potential users of these platforms, and more generally for those interested in PT operations. Certain findings may also inform designers and engineers of PT systems to help better understand real-world usage patterns and what features have the greatest impact on system usability.

In this study, these aspects are explored by extracting over five years of beam-level information from treatment log files and combining it with clinical/demographic information pulled from UFHPTI's oncology information system, MOSAIQ (Elekta, Stockholm, Sweden). The combination of these data streams allows for technical comparisons across anatomical sites and helps answer fundamental questions about how these PT systems are utilized. Patterns-of-use analyses have previously been reported for both multi-room and single-room centers, characterizing case mix, process times, and treatment capacity.^1^, ^2^ The present work extends that approach in 2 respects: it examines 2 distinct platforms operating concurrently within 1 facility, and it captures the delivery parameters that govern room selection, including beam geometry, energy, and range-shifter use, rather than treatment volume and timing alone. The resulting dataset provides a detailed cross-section of a complex PT operation.

## Methods and materials

### Data extraction

Machine log files were provided by the local service team for both systems and collected under a research protocol approved by UF IRB-01 (IRB202501587). As this study focused primarily on PBS, the rooms included for the P-PLUS were Gantry Room 2 (alias “GTR2”) and Gantry Room 1 (alias “GTR1”). The former added PBS capability in 2017, with log files provided from 2018 to 2024 (7 years); the latter was upgraded in 2023, with log files provided from mid-2023 to 2024 (1.5 years). The P-ONE, also known as “CGTR” based on its compact gantry, went live in late 2019, with log files provided from 2020 to 2024 (5 years). A custom Python script was written to extract the following information for each delivered beam: date, time, room, medical record number, beam name, gantry angle, couch angle, number of energy layers, total number of spots, delivery time, monitor units (MU), and dose. Beams were discarded if they contained missing information, labeled as “QA,” or delivered between the hours of 2:00 and 6:00 AM when no patient treatments occur. Additional information was extracted from the MOSAIQ SQL database including: date, medical record number, beam name, snout, range shifter, maximum energy, diagnosis code, date of birth, schedule note, and physician. The schedule note was particularly useful because it often described the anatomical site, allowing cross-reference with the diagnosis code, and indicated whether anesthesia was used or a device, such as a pacemaker, was present. The 2 data streams were combined using Excel’s (Microsoft, Redmond, WA) VLOOKUP function based on common information found within each data set. The merged data was processed using MATLAB R2023a (MathWorks, Natick, MA) to group and analyze the information in a variety of ways, for example, based on patient, anatomical site, day, month, and quarter. The final database included 120 367 delivered PBS beams for the P-PLUS and 95,587 for the P-ONE.

### Session time analysis

Treatment time per session was calculated by dividing the total treatment time, from the start of the first treatment to the end of the last, by the number of sessions treated that day. Recorded delays, including machine downtime and other documented interruptions, were subtracted from the daily total, such that the resulting value reflects productive treatment time. Days on which this value fell below 15 minutes or exceeded 100 minutes were excluded, a filtering approach consistent with prior analyses of proton treatment times.^3^ Notably, this measure represents an aggregate, encompassing patient setup, imaging acquisition and review, couch motion, and beam delivery without resolving these components individually.

Due to the fact that a PT treatment course typically spans several weeks, treatment time on consecutive days reflects largely the same patient cohort; daily values were accordingly autocorrelated (lag-1 autocorrelation 0.33-0.66 across rooms, decaying to zero over 17-39 treatment days, comparable to the length of a treatment course), meaning that treating individual days as independent observations would overstate the effective sample size. Daily values were therefore averaged within each calendar month, and the monthly mean was used as the unit of analysis. Pairwise differences in treatment time per session were evaluated using Welch's *t*-test, which does not assume equal variances. Three pairwise comparisons were performed (GTR2 vs GTR1, GTR2 vs CGTR, and GTR1 vs CGTR), with significance assessed using a Bonferroni-adjusted α of 0.0167. Comparisons were restricted to July 2023 through December 2024, during which all three rooms delivered PBS in routine clinical operation; GTR1's first month following clinical release was excluded as a start-up period.

Statistical analyses were performed in MATLAB R2023a. The analysis code was drafted with the assistance of Claude Opus 5 (Anthropic, San Francisco, CA) and independently re-implemented from a written specification by a second model (GPT 5.6 Sol, OpenAI, San Francisco, CA); both implementations returned identical test statistics when run in MATLAB. All code and results were reviewed and verified by the authors, who take full responsibility for the accuracy of the analysis.

### System parameters

Understanding the differences among the PBS treatment rooms included in this study (GTR1, GTR2, and CGTR) is important, as their utilization is highly dependent on their capabilities. As shown in Table 1, each nozzle differs in spot size and maximum field size. GTR2 has the largest spot size due to the lack of vacuum in the universal nozzle housing. GTR1 and CGTR both use dedicated nozzles with full vacuum, yielding similar spot sizes; however, CGTR’s maximum spot size is larger because the P-ONE can reach a lower minimum energy. The maximum field size is largest in GTR2, similar in GTR1 except at the highest energies where older scanning power supplies limit the maximum deflection angle, and smallest in CGTR.

**Table 1:** Characteristics of the Gantry Treatment Rooms 1 and 2 (GTR1/GTR2) of the ProteusPLUS and the Compact Gantry Treatment Room (CGTR) of the ProteusONE.

Room designation	GTR1	GTR2	CGTR
Nozzle type	Dedicated - PBS	Universal - PBS	Dedicated - PBS
Spot size - σ (mm)	2.9-5.1	4.6-11.0	3.4-7.8
Max field size (W cm x L cm)	30 (24 @ max E) x 40	30 x 40	20 x 24
Min spot (MU/fx)	0.025		0.012
Layer switching time (s)	2-3		<1
Cyclotron	C230		S2C2
Max E (MeV)	227		227
Min E (MeV)	98		70
Range shifter			
Thickness (cm)	6.5		3.5
Density (g/cm^3^)	1.2		1.2
WET (cm)	7.4		4.0
Moveable tray	Yes^a^	No	Yes^a^
Gantry rotation (deg)	360		220 (−35 to 185)
Patient positioning system	Schaer (6DoF)		Leoni (6DoF)
Rotation (deg)	90-270 (through 0)		−5 to 185 (through 270)
CBCT			
Scan type	Half scan / full scan		Half Scan / dual half scan
Axial FOV (cm)	34		26 or 50
Scan length (cm)	34		26
Min scan time (s)	Low speed > 80-140, High speed > 40-70		Low speed > 86-198, High speed > 52-131
Planar imaging	Orthogonal kV (2 panels)		Orthogonal kV (1 panel), fixed oblique kV (2 panels)

DoF, Degrees of Freedom; PBS, Pencil Beam Scanning; WET, Water Equivalent Thickness.

a The larger, primary snout has a moveable tray, while smaller snout attachments use a fixed slot for a range shifter.

Additional differences are inherent to the design of each platform. The rooms associated with the P-PLUS have full gantries with 360° of rotation, whereas the P-ONE includes a compact gantry with 220° of rotation and relies on its robotic couch to flip the patient 180° (head first supine to feet first supine) to treat both left and right lateral beams, that is, couch flip. The “patient positioning system” listed in Table 1 describes the couch vendor; Schaer for the P-PLUS, with a traditional rotational swing of 90° to 270°, and Leoni for the P-ONE, with the aforementioned ability to “flip” the couch from −5° to 185°. Note that all fields in a treatment plan are delivered during each session; a “one side per day” beam arrangement is not used. The P-PLUS has longer layer switching times, though they have been improved compared to the original installation.^4^ The P-PLUS also has a thicker range shifter, equivalent to 7.4 cm in water, due to its higher minimum energy. Comparatively, the range shifter on the P-ONE has water equivalence of 4.0 cm. The range shifter in GTR2 requires manual attachment and removal. The range shifters for the large snout of GTR1 and CGTR utilize a moveable tray that can be interlocked electronically, which is safer and more efficient. The smaller snouts still require manual attachment and removal in those rooms. The use of variable range shifters is discouraged by standard practice, for example, all fields should use either a range shifter or no range shifter. Finally, the imaging configurations are different between the 2 systems with the P-PLUS utilizing 2 orthogonal panels for 2D kV imaging with one of those panels used for CBCT, whereas the P-ONE utilizes one panel for 2D kV imaging and CBCT, and additionally supports oblique kV imaging with 2 fixed panels. Of note, UFHPTI’s P-PLUS utilizes second-generation system architecture; its specifications will differ in some respects from newer installations. Other differences between the P-PLUS and P-ONE, for example, power consumption, physical size, shielding requirements, etc., are outside the scope of this study.

Room assignment is made during treatment planning and is guided primarily by field size and spot size requirements. Treatment sites that frequently require a field size larger than 20 cm × 24 cm are assigned to GTR2 or GTR1. Moving targets in the thorax and abdomen are assigned to GTR2, where the large spot helps mitigate the interplay effect. Pediatric patients and those requiring anesthesia are preferentially scheduled in CGTR. Remaining cases are assigned based on availability and scheduling considerations. GTR1 is also typically staffed for 2 therapist shifts per day compared with three in GTR2 and CGTR, so its daily patient volume is constrained by operating hours rather than by demand or room capability.

## Results

During the timeframes explored in this study, the mean daily patient volumes were 23.9 ± 6.8 for GTR2 (max = 48), 22.6 ± 5.3 for GTR1 (max = 35; staffed for 2 shifts rather than three), and 27.6 ± 7.1 for CGTR (max = 45). The average treatment times per session varied by year with the annual totals described fully in Table S1. When restricted to the final 18 months during which all three rooms utilized PBS, mean daily patient volumes were 27.4 ± 5.4 for GTR2 (max = 48), 22.9 ± 5.1 for GTR1 (max = 33), and 27.0 ± 4.6 for CGTR (max = 44). The mean treatment time per session during the 18 months span was 25.6 ± 1.7 minutes in GTR1, 28.2 ± 2.5 minutes in GTR2, and 26.9 ± 2.4 minutes in CGTR (mean ± SD of 18 monthly means), Table S2. GTR2 required significantly more time per session than GTR1 (difference 2.7 minutes; 98.33% CI 0.9-4.5; *P* = .0007). Neither GTR2 vs CGTR (1.4 minutes; 98.33% CI −0.7 to 3.4; *P* = .099) nor GTR1 vs CGTR (−1.3 minutes; 98.33% CI −3.1 to 0.5; *P* = .076) reached the adjusted significance threshold, Appendix A.

The anatomical case mix per year is listed in Figure 1 (also includes Gantry Room 3, alias “GTR3,” of the P-PLUS, which was never upgraded from passive scattering and stopped being utilized for patient treatments in 2024). As shown, GTR2 was the first to receive PBS capability and initially treated cancers found within a variety of sites, including the head and neck (H&N, 34%), breast (20.4%), prostate (11.2%), brain (10.2%), pelvis (8.7%), central nervous system, including the spinal column and base of skull (CNS, 7.8%), and skeletal anatomy (bone, 5.8%). The case mix narrowed in the second year, with only sites inclusive of the H&N, breast, prostate, and brain having more than a 5% share. With the addition of a P-ONE in 2020, a significant fraction of H&N, brain, and CNS cases moved to this system, while GTR2 continued to treat prostate, breast, and some H&N and pelvis cases. At the time, moving targets in the thorax were still treated using passive scattering in GTR3, but with the phase out of that modality between 2022 and 2024, these were transitioned to GTR2 with its universal nozzle and larger spot size. By 2024, the facility operated three PBS gantries, with GTR2 mainly treating prostate, breast, and thorax cases, and GTR1 and CGTR treating prostate, H&N, brain, and CNS cases (with a slightly higher percentage of brain and CNS in CGTR). The percentage of patients receiving PBS by year from 2018 to 2024 was 21%, 26%, 60%, 74%, 78%, 91%, and 100%. The overall case mix for the facility remained largely consistent between 2019 and 2024 (Figure 2).

**Figure 1:**
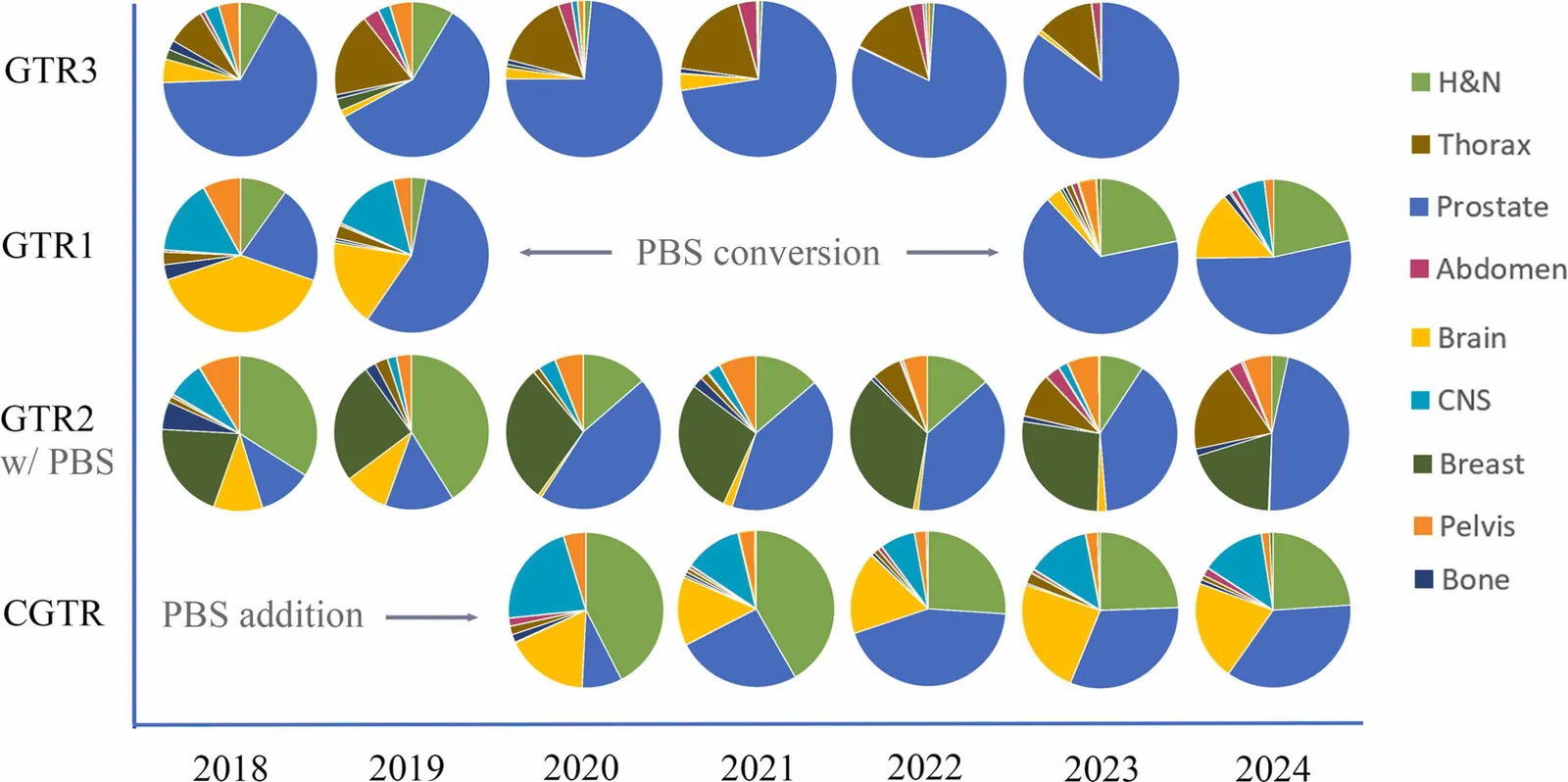
Pie charts showing the anatomical case mix for Gantry Treatment Rooms 1, 2, and 3 (GTR1, GTR2, GTR3) of the ProteusPLUS, and the Compact Gantry Treatment Room (CGTR) of the ProteusONE between 2018 and 2024. GTR2 was upgraded with pencil beam scanning (PBS) in 2017. CGTR came online with PBS in 2020. GTR1 was upgraded with PBS in mid-2023. GTR3 was never upgraded from passive scattering and has been inactive since 2024.

**Figure 2:**
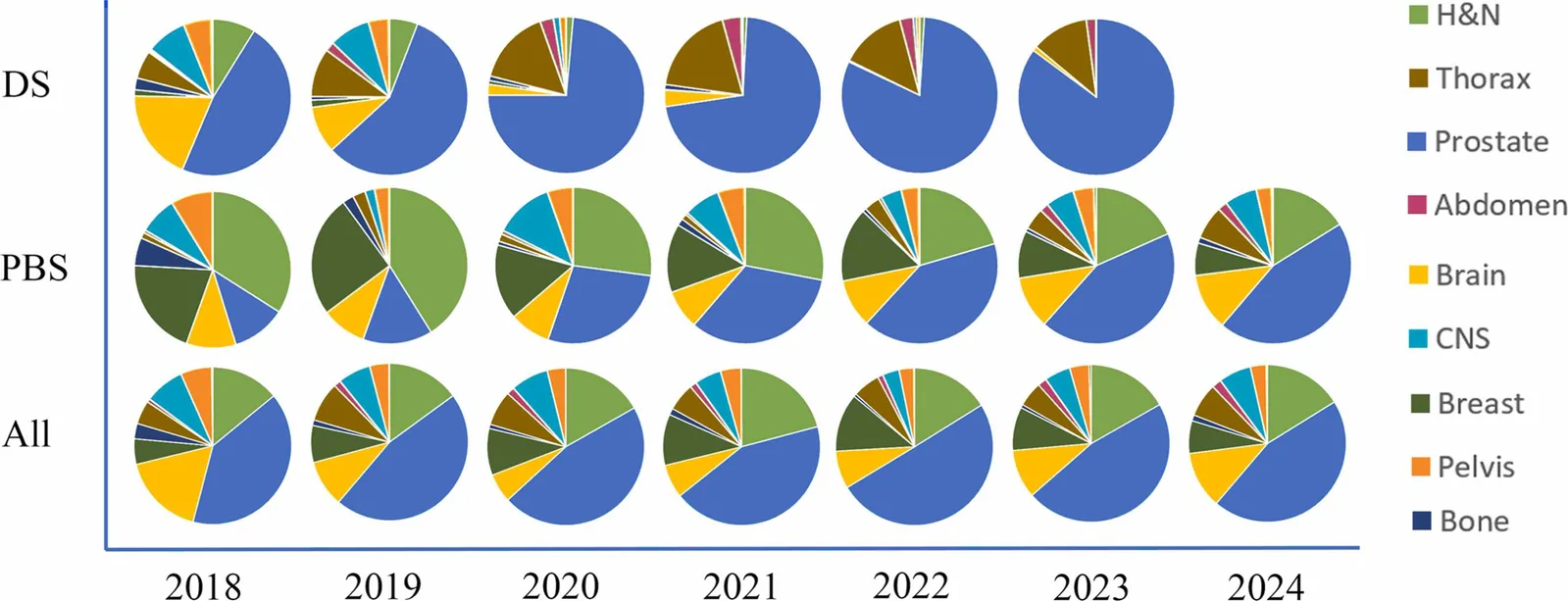
Pie charts showing the anatomical case mix treated with passive scattering (PS) and pencil beam scanning (PBS) between 2018 and 2024, as well as the overall proton case mix during this timeframe.

Looking closely at each anatomical site, Table 2 provides the number of patients, treatments, beams, and beams per treatment, as well as the percentage of treatments involving a range shifter, anesthesia, pediatric patient (age < 18), or couch flip (P-ONE only where the couch rotates from 0° to 180°) when using PBS. As seen in the table, the average number of beams per treatment was similar across treatment rooms for the same anatomical site, though brain, CNS, and H&N cases showed slightly higher average values for CGTR and GTR1 compared to GTR2. Looking specifically at H&N cases, Figure 3 provides polar histograms of all PBS beams treated in these rooms, binned by gantry and couch angle. For GTR2 and GTR1, common angles included 0° (as used with a three-beam, inverted Y technique), 180° (as used with a three-beam, Y technique), and oblique angles found within specific windows of each quadrant. Couch angles remained at 0° ± 15°. The impact of the P-ONE’s partial gantry is clearly seen in CGTR, where gantry angles are limited to 2 oblique quadrants, as well as 0° and 180° couch angles consists of both 0° and 180°, that is, includes a couch flip, with some minor variation. Table 2 shows that 70.4% of H&N treatments on the P-ONE used a couch flip, a rate comparable to brain (71.7%), abdomen (75.6%), and pelvis (76.7%) cases. By contrast, nearly all prostate cases used a couch flip, while none of the breast cases did.

**Table 2:** Number of patients, treatments, beams, and beams per treatment, as well as the percentage of treatments involving a range shifter, anesthesia, pediatric patient (age < 18), or elbow change for Gantry Treatment Room 2 (GTR2) and Gantry Treatment Room 1 (GTR1) of the ProteusPLUS and the Compact Gantry Treatment Room (CGTR) of the ProteusONE when treating with pencil beam scanning (PBS).

ProteusONE - CGTR	Prostate	Head/Neck	Brain	Breast	Bone	Thorax	Abdomen	CNS	Pelvis	Other	Total
No. patients	382	375	236	7	25	30	28	171	44	6	1304
No. treatments	10 443	10 215	6291	55	391	447	394	4325	953	128	33 642
No. beams	21 751	33 580	19 005	110	1104	1411	1347	14 557	2333	389	95 587
No. beams per treatment	2.07	3.12	3.01	2.00	2.72	3.12	3.38	3.31	2.42	3.04	2.84
Percentage beams w/											
Range Shifter	1.8%	99.3%	68.4%	100.0%	91.8%	99.8%	95.5%	79.4%	68.0%	92.0%	67.0%
Percentage treatments w/											
Range Shifter	1.5%	99.2%	68.3%	100.0%	88.7%	99.3%	92.4%	76.8%	65.8%	88.3%	59.0%
Anesthesia	0.6%	3.3%	26.0%	0.0%	24.3%	38.9%	68.0%	19.1%	29.3%	0.8%	10.9%
Pediatric	0.3%	8.2%	56.8%	0.0%	65.5%	63.5%	82.5%	60.6%	53.2%	43.8%	25.2%
Couch flip	99.2%	70.4%	71.7%	0.0%	61.4%	52.6%	75.6%	51.7%	76.7%	80.5%	77.0%
*ProteusPLUS - GTR2*											
No. patients	496	252	59	433	28	119	18	55	97	1	1558
No. treatments	14 576	6508	1162	10 809	697	2569	447	966	2485	15	40 234
No. beams	30 250	21 026	3341	23 116	1812	8495	1367	2895	5926	60	98 288
No. beams per treatment	2.07	2.96	2.80	2.04	2.59	3.30	2.92	2.80	2.34	4.00	2.44
Percentage beams w/											
Range Shifter	16.6%	99.4%	96.0%	99.6%	88.0%	97.6%	65.3%	89.6%	71.9%	100.0%	71.1%
Percentage treatments w/											
Range Shifter	16.7%	99.4%	96.1%	99.6%	88.0%	96.9%	58.2%	92.1%	74.0%	100.0%	66.8%
Anesthesia	0.0%	1.8%	25.6%	0.0%	0.0%	0.0%	2.7%	5.0%	0.8%	0.0%	1.2%
Pediatric	0.0%	9.7%	52.2%	0.6%	7.2%	25.7%	29.5%	30.0%	16.5%	0.0%	7.1%
*ProteusPLUS - GTR1*											
No. patients	179	72	51	2	5	5	7	16	10	1	348
No. treatments	5015	1873	991	20	99	57	118	374	230	27	8804
No. beams	10 217	6127	2987	40	336	159	326	1192	615	80	22 079
No. beams per treatment	2.03	3.26	3.01	2.00	3.39	2.70	2.76	3.19	2.60	2.96	2.51
Percentage beams w/											
Range Shifter	8.1%	100.0%	96.5%	100.0%	51.2%	100.0%	96.3%	100.0%	70.9%	100.0%	55.4%
Percentage treatments w/											
Range Shifter	8.0%	100.0%	96.4%	100.0%	58.6%	100.0%	95.0%	100.0%	73.9%	100.0%	46.0%
Anesthesia	0.0%	0.1%	2.1%	0.0%	1.0%	3.5%	2.5%	2.7%	3.5%	0.0%	0.5%
Pediatric	0.0%	2.1%	26.0%	0.0%	1.0%	33.3%	43.2%	23.0%	23.0%	0.0%	5.8%

Data is inclusive of the following dates per room; GTR2, 2018-2024; CGTR, 2020-2024; GTR1, 2023-2024.

**Figure 3:**
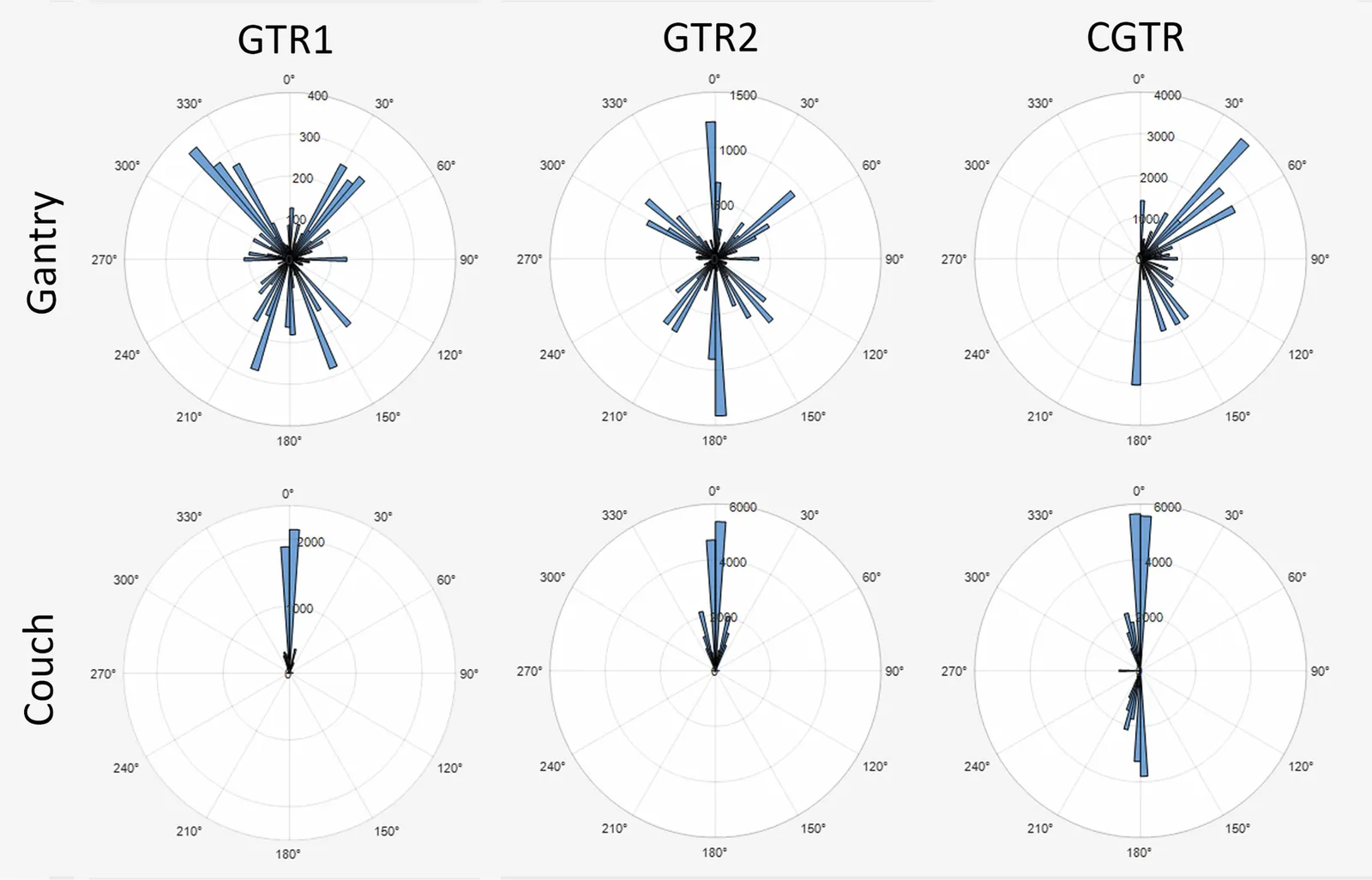
Polar histograms of gantry and couch angles used for head and neck treatments in Gantry Rooms 1 and 2 (GTR1 & GTR2) of the ProteusPLUS and Compact Gantry Room (CGTR) of the ProteusONE.

Notably, the data shows that the use of four oblique beams for H&N cases was rare prior to 2022, after which the technique comprised roughly 30% of all cases across all rooms. This explains why 0° and 180° beams, which are associated with the three-beam, Y and inverted Y techniques, appear more prevalent in GTR2, which began treating with PBS much earlier than either CGTR or GTR1. It also explains why the average number of beams per treatment is slightly higher in CGTR and GTR1 compared to GTR2. Polar histograms for other anatomical sites, for example, brain, prostate, and breast, can be found in Figures S1–S4.

Also seen in Table 2, the case mix on the P-ONE included a higher percentage of pediatric patients per treatment, 25.2%, compared to the P-PLUS, 7.1% (GTR2) and 5.8% (GTR1) when using PBS. Table S4 provides a further breakdown of the number of pediatric and anesthesia cases per day as averaged across each month. For the P-ONE, the mean number of pediatric and anesthesia cases per day was 6.7 ± 2.4 and 2.7 ± 1.8 (approx. 40% of pediatric cases done with anesthesia). The maximum number of pediatric and anesthesia cases per day as averaged across a month was 14.5 and 8.4, respectively. For the P-PLUS, the number of pediatric cases was similar between the 2 PBS rooms at 1.2 ± 1.0. Furthermore, the data shows that anesthesia was rarely used when these cases were treated on the P-PLUS, likely because patients needing anesthesia are typically grouped together on the P-ONE for better workflow efficiency. Note that prior to 2020 and the addition of the P-ONE, the majority of pediatric patients were still treated with passive scattering in GTR3 and GTR1 (Table S5).

Considering the use of range shifters, Figure 4A and B present absolute histograms of all delivered beams, showing how range-shifter usage relates to maximum beam energy for the P-PLUS’s GTR2 and the P-ONE’s CGTR. As seen, the cyclotron’s highest energy is used far more often in GTR2 (7.5% compared to 0.4% in CGTR), while overall range shifter usage per beam is similar (71.1% compared to 67.0%). In GTR2, the most common ranges of maximum beam energies were 225 to 227 MeV and 197 to 199 MeV; in CGTR they were 195 to 197 MeV and 143 to 145 MeV. In GTR2, use of the maximum cyclotron energy strongly correlates with the use of a range shifter, reflecting beams that combine deep and shallow targets—most commonly prostate (deep) and pelvic nodes (shallow), that is, PSVN (Figure 4C). In CGTR, similar cases seldom need a range shifter because the P-ONE’s lower available cyclotron energies allow the beam to effectively treat the pelvic nodes without it (Figure 4D). When restricted to prostate-only treatments, the histograms for the 2 systems look much more alike (Figure 4E and F). For sites superior to the pelvis and abdomen—brain, breast, CNS, head and neck, and thorax—82% and 49% of beams delivered in GTR2 had maximum energies below 200 and 180 MeV, respectively (Figure 4G); in CGTR these fractions increased to 97% and 79% (Figure 4H). In these supra-abdominal cases, fewer beams required a range shifter in CGTR than in GTR2 (86.6% vs 98.5%).

**Figure 4:**
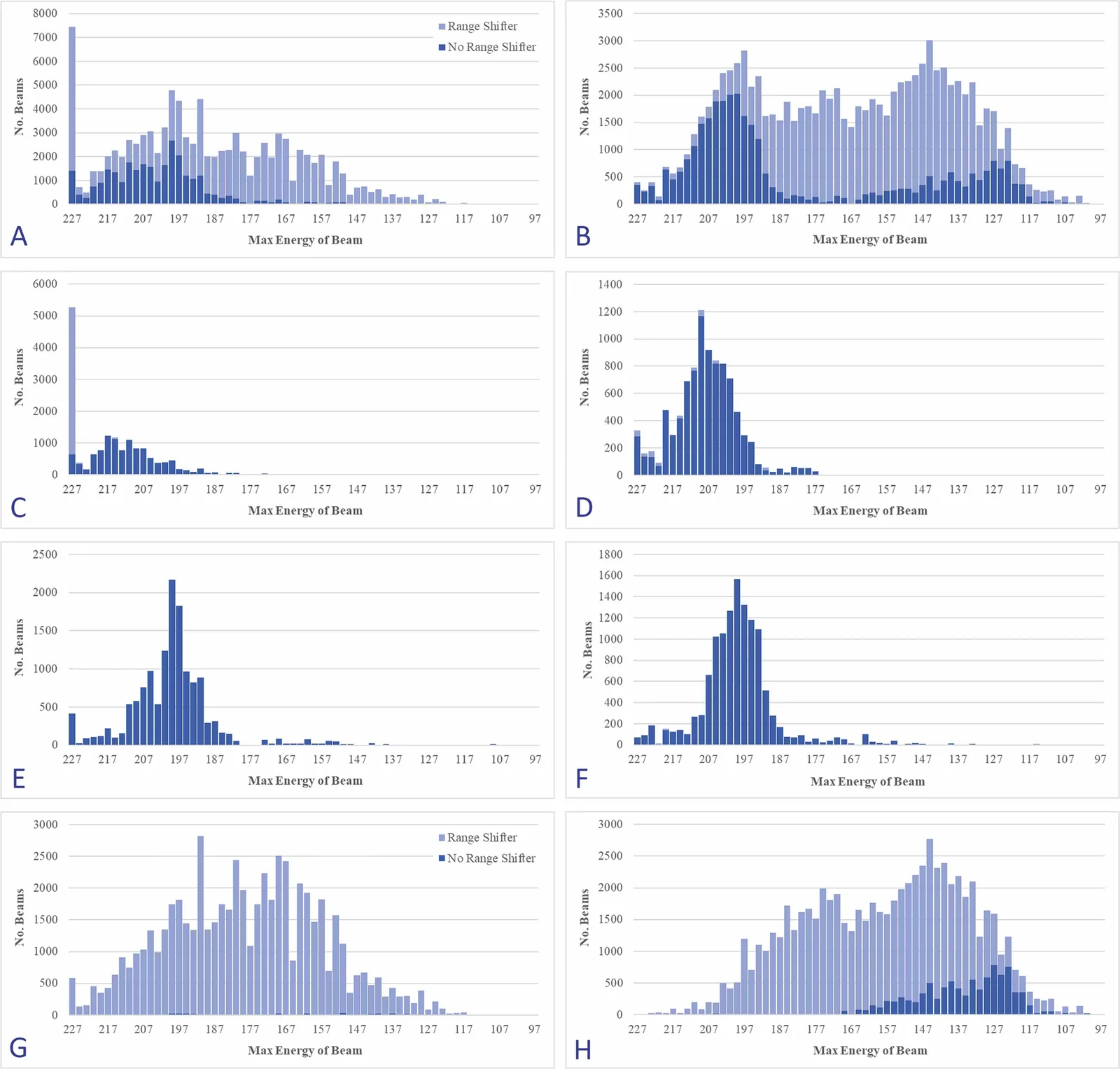
Histograms of treatment-beam counts binned by maximum energy. Light blue indicates beams delivered with a range shifter; dark blue indicates beams delivered without a range shifter. The first column (A, C, E, G) shows beams from Gantry Treatment Room 2 (GTR2) of the ProteusPLUS, and the second column (B, D, F, H) shows beams from the Compact Gantry Treatment Room (CGTR) of the ProteusONE. Panels A and B include all anatomical sites; panels C and D include prostate, seminal vesicle, and pelvic nodal irradiation (PSVN); panels E and F include prostate-only treatments; panels G and H include treatments of the head and neck, brain, breast, central nervous system, and thorax.

## Discussion

The introduction of PBS through both system upgrades and facility expansion substantially changed clinical utilization patterns at UFHPTI. This is clearly evident in Figure 1, Figure 2 where the distribution of anatomical sites changed each time a new PBS room was added. The initial experience in year 1 (2018) shows diversity among treatment sites, which narrowed somewhat in year 2. In these early years, the program focused on cases that were challenging to treat with passive scattering or that offered clear dosimetric advantages with PBS. Two anatomical sites that fit this criterion were H&N and breast, where PBS eliminated the need for complex beam matching, reduced skin dose and associated toxicities, and for H&N specifically, enabled more complex dose distributions via multi-field optimization.^5^ PBS also enabled treatment of advanced prostate cancer involving the pelvic lymph nodes. In year 1, only 26% of PBS-treated prostate cases included pelvic nodal irradiation, but in GTR2, this rose to 60% and 61% in years 2 and 3 (Table S6). Across the study period, GTR2, with its larger field size, consistently had the highest proportion of nodal cases. Notably in 2024, CGTR showed a comparable share of nodal cases (60% for GTR2 vs 57% for CGTR), suggesting that the 20 cm × 24 cm field size on the P-ONE is adequate for the majority of such cases.

Due to the completion of a contract in late 2018 with the National Health Services, which provided PT to pediatric patients from the United Kingdom (UK), the rollout of PBS largely coincided with a decrease in the number of pediatric cases treated at UFHPTI. This partially explains the decrease in CNS and bone (ie, sarcoma) cases treated in year 2 with PBS and also the overall decrease in the percentage of brain cases seen between 2018 and 2019 (Figure 2). After the addition of the P-ONE in 2020, the data shows a clear preference for treating pediatric patients on this platform. This was mainly due to the system’s smaller spot size (compared with GTR2), which enabled tighter dose distributions and reduced dose to nearby critical structures. In addition, faster layer switching and quicker image acquisition via oblique imaging were assumed to shorten overall treatment times. Finally, room-specific features such as the Philips Ambient package (Koninklijke Philips N.V., Amsterdam, Netherlands) made the space more welcoming for pediatric patients. With the addition of PBS via a dedicated nozzle in GTR1 in 2023, the spot sizes are now largely comparable between GTR1 and CGTR. Regardless, the data shows a continued preference for treating pediatric patients on the newer platform.

As access to PBS expanded in 2020 and again in 2023, the range of cases treated with passive scattering decreased substantially (Figure 2). After the remaining H&N cases and many brain/CNS cases shifted to PBS between 2019 and 2020, passive scattering was reserved mainly for gland-only prostate treatments and for moving targets in and around the thorax and abdomen. Due to concerns about the interplay effect and inadequate robustness when using PBS, anatomical sites associated with significant motion were the last to transition to this modality. With more experience and as better planning tools and techniques developed, for example, multi-phase optimization and volumetric repainting, these cases were moved to PBS, almost exclusively in GTR2, where the larger spot size helps mitigate interplay.^6^ Another site that showed a strong preference for GTR2 was breast, with 433 patients treated compared to only 7 in CGTR. Two factors drove this preference. First, the larger spot size (and resulting increased lateral penumbra) is less problematic for breast treatments because most critical structures lie distal to the target. Second, the larger field size allows cases involving regional lymph nodes to be covered with a single-isocenter plan. It is unclear, however, what fraction of such cases would require multiple isocenters on the P-ONE, since the available data cannot distinguish between room selection driven by technical constraints and selection driven by historical practice (i.e., whether patients were treated in the GTR2 due to field size limitations in CGTR or simply because we are in the habit of treating these patients in that room).

As noted previously, by 2024 the conversion was complete, with GTR2 mainly treating prostate, breast, and thorax cases, and GTR1 and CGTR treating prostate, H&N, brain, and CNS cases. CGTR had a slightly higher share of brain and CNS cases due to the aforementioned preference for treating pediatric patients on the P-ONE. Over the final 18 months, when all three rooms were configured with PBS, mean treatment time per session was 1.3 minutes longer in CGTR than in GTR1, a difference that did not reach the adjusted significance threshold. This is notable given the difference in case mix between them: pediatric cases, particularly those requiring anesthesia, take longer to treat, and the majority of these were treated in CGTR. Our standard scheduling allotment for craniospinal irradiation under anesthesia is 45 minutes, compared with 30 minutes for a head and neck case and 15 minutes for a gland-only prostate case. Anesthesia has separately been reported to add approximately 13 minutes of in-room time for pediatric craniospinal irradiation using PBS.^7^ During the 18-month span, 32.0% of CGTR treatments involved pediatric patients and 18.1% involved anesthesia, compared with 5.8% and 0.5% in GTR1. Applying the reported anesthesia penalty to this difference alone would predict roughly 2.4 minutes of additional session time in CGTR before accounting for the longer setup and positioning times associated with pediatric cases in general. That the observed difference is smaller than this suggests that CGTR absorbed a more complex case mix without a proportionate increase in session time.

Further evidence of available capacity comes from a multi-room downtime event previously reported by our group, during which CGTR sustained an average of 18.4 min per session across a case mix comparable to that of GTR1 in 2024, with approximately 50% of the anatomical mix being prostate cases.^8^ That value was calculated without subtracting delays and is therefore a conservative estimate relative to the values reported here. The event involved extended operating hours and modified imaging protocols and does not represent routine operation, but it demonstrates that session times well below those observed in steady-state practice are achievable on this platform. While the present data cannot identify which aspects of the system or workflow account for these observations, faster layer switching and oblique kV image acquisition are plausible contributors, and quantifying their respective effects will require the component-level analysis planned in our next study.

Relatedly, 77% of all treatments on the P-ONE required a couch flip, yet over the final 18 months, when all three rooms were configured with PBS, CGTR sustained a daily patient volume comparable to the busiest P-PLUS room (27.0 vs 27.4 sessions/day), suggesting that the additional step is not prohibitive at the operational level. Notably, the data also indicates that there were a number of times in which a couch flip could have been avoided. Among H&N beams delivered on the P-ONE, only 2% used a gantry angle of −35° to −5° with the couch near 0° to achieve a right anterior oblique beam, even though an additional 7% could have used that setup but instead were delivered at gantry angles of 5° to 35° with the couch near 180°. Considering all beams, 1% used −35° to −5° with the couch near 0°, while another 3.5% were eligible for that configuration. It is not clear that this alternative setup would have been appropriate in every case—for example, the couch may have been placed near 180° to accommodate other beams (such as a right lateral beam). Even so, these findings suggest a small but actionable opportunity to further optimize beam delivery for select cases when using the P-ONE.

Lastly, the relationship between range shifter usage, minimum cyclotron energy, and maximum beam energy is an interesting finding. As an illustration, Figure S6 shows a PSVN case with a minimum depth of 4 cm to the ipsilateral pelvic nodes and a maximum depth of 24 cm to the prostate and contralateral pelvic nodes. Because the minimum depth is less than the range provided by the minimum cyclotron energy available on the P-PLUS, a range shifter is needed. For this system, we utilize a single range shifter with an approximate range pull back of 7.4 cm. This means that the maximum depth for the ipsilateral beam treating the contralateral side near the level of the prostate becomes 31.4 cm, requiring the maximum energy of the cyclotron. On the P-ONE, however, spots can be placed at a depth of 4 cm without using a range shifter, and only 195 MeV is needed to reach the 24 cm depth. Thus, as this example (and the histograms in Figure 4) illustrates, reducing the minimum energy of a cyclotron from 98 to 70 MeV also reduces the need for a range shifter, and consequently, lowers the maximum energy required for the vast majority of PSVN cases. Such cases represent a significant fraction of those requiring the maximum energy of the cyclotron at our institution. Alternatively, providing a thinner range shifter would also reduce the need for maximum beam energy on the P-PLUS. In this example, a 4 cm range shifter yields a maximum depth of 28 cm and a maximum beam energy near 210 MeV.

Notably, this example reflects our institutional convention for treating PSVN cases, in which the ipsilateral beam treats through to the contralateral side where the targets are contiguous and central, such as nodal volumes at the level of the prostate or where they converge along the spine; where the pelvic nodes separate laterally, a midline blocking structure restricts each beam to the ipsilateral volume. Conventions vary across centers, and more extensive midline blocking could lower the need for the maximum energy of the cyclotron when treating this site.

Based on our experience, very few, if any, patients have been declined PBS based on the inability of the beam to reach a deep-seated target within a large patient; denials more often result from the 350 lb. weight limit of the couch, which exists for both platforms. With that in mind, it is an interesting thought experiment to consider how lowering the maximum cyclotron energy could affect cyclotron size, shielding, energy consumption, and patient eligibility. Across all beams, only 9.5% in GTR2 and 1.2% in CGTR exceeded 220 MeV. If we further consider lowering the C-230’s minimum energy to reduce the reliance on a range shifter, the share above 220 MeV in GTR2 could drop to 2.4%, the rate observed in GTR2 cases without a range shifter. Relative to a 230 MeV design, reducing the maximum energy to 220 MeV could shrink the cyclotron diameter and reduce neutron production by an appreciable amount. Energy consumption would also decrease, though precise savings require further study.

These results can be compared to simulation-based studies that have suggested limits of 207 MeV in order to treat 95% of all cases when using proton arcs,^9^ or 180 MeV to treat the prostate (without nodes) when using equally weighted discrete beam angles, that is, discrete arc.^10^ In these studies, the use of a range shifter is excluded or not described, and the multi-angular approach reduces the reliance on deep, lateral beams, as currently used when treating the prostate. These methodological differences likely account for the higher values observed in the present study, though the difference is modest: 220 MeV covers 98% to 99% of cases here, whereas Sengbusch et al, analyzing 100 patients, report 207 MeV for 95%. Whereas the prior estimates derive from selected planning cohorts under idealized geometry, the present figures reflect every beam actually delivered over the study period, and therefore capture the full range of anatomy, beam arrangements, and range-shifter use encountered in routine practice. On that basis, 220 MeV should be regarded as a conservative bound grounded in delivered treatments rather than a theoretical minimum. Overall, these data suggest that a 220 MeV ceiling could have minimal impact on patient eligibility while enabling a slightly smaller, more efficient cyclotron, subject to confirmation by detailed design and shielding analyses.

This study has a number of limitations. First, results are based on the experience of a single institution, which may limit the generalizability of these findings to other centers with different hardware configurations, workflows, staffing models, patient populations, or referral patterns. Second, the room-level comparisons are observational and reflect clinical allocation decisions rather than randomized assignment; thus, differences in throughput, case mix, pediatric utilization, and beam characteristics may be influenced by historical practice patterns, provider preferences, and evolving institutional protocols in addition to intrinsic platform capabilities. Third, some important questions could not be answered definitively from the available dataset, including the extent to which breast or other cases were assigned to particular rooms because of true technical constraints versus established practice patterns.

## Conclusions

This study combines more than five years of beam level data with clinical and demographic records to provide a rare, real-world analysis of how 2 IBA PT platforms (P-PLUS and P-ONE) are used within a single, high-volume center. It also documents UFHPTI’s stepwise transition to PBS through system upgrades and expansion, showing that expanded PBS capacity substantially reshaped case allocation over time. Room selection was driven primarily by differences in spot size and field of view, and secondarily by imaging configuration, layer-switching performance, and pediatric-oriented features such as the Philips Ambient package.

Consistent with these differences, GTR2, using a universal nozzle with larger spot size and larger field size, was used predominantly for prostate (including a high proportion of nodal cases), breast, and thorax cases (including moving targets), while GTR1 and CGTR, using dedicated nozzles with smaller spot sizes, were used primarily for prostate, H&N, brain, and CNS cases. CGTR also showed a clear preference for pediatric and anesthesia-supported workflows, reflecting its smaller spot size along with system features expected to reduce treatment time, including faster layer switching and fixed oblique kV panels that require no deployment between fields. Based on the observed case mix, the P-ONE’s smaller field size does not appear to be a major limitation for PSVN cases, but it may necessitate multiple isocenters for some breast treatments (though this is not answered definitively with the available data). Likewise, couch flips on the P-ONE do not appear to meaningfully limit the efficiency of the system for handling a high patient volume. Finally, an analysis of range shifter and beam energies shows that a lower minimum energy reduces range-shifter dependency and the associated need for maximum cyclotron energy, particularly for PSVN cases. Furthermore, a maximum cyclotron energy of 220 MeV might be suitable for upwards of 98% of patients while enabling certain design tradeoffs related to cyclotron size, shielding, and energy consumption.

## CRediT authorship contribution statement

Perry Johnson: Conceptualization, data curation, formal analysis, writing – original draft. Twyla Willoughby: Methodology, data curation, formal analysis, writing – review and editing. Jonathan Alvarez: Methodology. Julien Forthomme: Methodology, writing – review and editing. An Van den Bulck: Methodology, writing – review and editing. Nancy Mendenhall: Writing – review and editing.

## Ethics declaration

The authors have NOT obtained informed consent from participants or their legal representatives. This is a retrospective chart review - the study was deemed exempt by the IRB.

This study was performed in compliance with relevant laws, regulatory frameworks, and guidelines where the research took place. The University of Florida - IRB 01 granted an exemption. The authors provided the following explanation: This is a retrospective chart review.

## Declaration of Generative AI and AI-Assisted Technologies in the Writing Process

During the preparation of this work the author(s) used Claude Opus 5 (Anthropic, San Francisco, CA) in order to provide minor editing and suggestions of alternative phrasing. Additionally, statistical analyses were performed in MATLAB R2023a where the analysis code was drafted with the assistance of Claude Opus 5 and independently re-implemented from a written specification by a second model (GPT 5.6 Sol, OpenAI, San Francisco, CA); both implementations returned identical test statistics when run in MATLAB. All code and results were reviewed and verified by the authors, who take full responsibility for the accuracy of the analysis.

## Funding

This work received funding from Ion Beam Applications (IBA – Louvain-la-Neuve, Belgium).

## Relationships

There are no additional relationships to disclose.

## Patents and Intellectual Property

There are no patents to disclose.

## Other Activities

There are no additional activities to disclose.

## Declaration of Conflicts of Interest

The authors declare the following financial interests/personal relationships which may be considered as potential competing interests: Perry Johnson reports financial support was provided by Ion Beam Applications SA. Perry Johnson reports a relationship with Ion Beam Applications SA that includes: funding grants. Both Perry Johnson and Nancy Mendenhall serve in editorial capacities for IJPT. Both Julien Forthomme and An Van den Bulck were employed by Ion Beam Applications SA during this study.

## Footnotes

Appendix A Supplementary data associated with this article can be found in the online version at doi:10.1016/j.ijpt.2026.101969.
