# Supplementary material for: A Comprehensive Beam-Level Assessment of Pencil-Beam Scanning Migration and Utilization at a Multi-System Proton Therapy Center
Source: Int J Part Ther. 2026 Oct 6;22:101969. doi: 10.1016/j.ijpt.2026.101969 (PMC13682380; doi:10.1016/j.ijpt.2026.101969)
Supplement: Supplementary file 1 [file mmc1.docx]

**Supplementary Material**

**Table S1.** Average time per session (minutes) by treatment room and year, calculated from observations retained after excluding outliers (days with mean values less than 15 or more than 100 minutes per patient); blank cells indicated years in which PBS was unavailable. Note that for GTR1, 2023 represents data averaged over half the year.

|  | **2018** | **2019** | **2020** | **2021** | **2022** | **2023** | **2024** |
| --- | --- | --- | --- | --- | --- | --- | --- |
| GTR2 | 41.70 | 41.10 | 36.20 | 34.20 | 28.40 | 27.90 | 28.90 |
| CGTR |  |  | 32.40 | 27.90 | 23.90 | 25.80 | 27.10 |
| GTR1 |  |  |  |  |  | 27.20 | 25.50 |

**Table S2.** Treatment time per session (minutes) by treatment room, July 2023–December 2024. Mean ± SD and range are computed across the 18 monthly means used as the unit of analysis; session counts are totals for the period.

| **Room** | **Months** | **Sessions** | **Sessions/Day** | **Time/Session** | **Monthly range** |
| --- | --- | --- | --- | --- | --- |
| GTR1 | 18 | 8,301 | 22.9 | 25.6 ± 1.7 | 22.0 - 29.3 |
| GTR2 | 18 | 9,126 | 27.4 | 28.2 ± 2.5 | 24.2 - 31.8 |
| CGTR | 18 | 9,865 | 27.0 | 26.9 ± 2.4 | 22.9 - 31.1 |

**Table S3.** Pairwise differences in treatment time per session between rooms, July 2023–December 2024. Welch's t-test on monthly means; the 98.33% confidence interval corresponds to the Bonferroni-adjusted α of 0.0167 for three comparisons. Positive values indicate longer times in the first-named room.

| **Comparison** | **Difference** | **98.33% CI** | **t (df)** | **p** |
| --- | --- | --- | --- | --- |
| GTR2 vs GTR1 | +2.68 | 0.89 to 4.47 | 3.78 (30.5) | 0.0007 |
| GTR2 vs CGTR | +1.38 | -0.67 to 3.43 | 1.70 (34.0) | 0.099 |
| GTR1 vs CGTR | -1.29 | -3.08 to 0.49 | -1.84 (30.7) | 0.076 |

**Table S4.** Mean, standard deviation (SD), maximum and minimum number of pediatric and anesthesia cases per day as averaged across each month.

|  | **Anesthesia** | | |  | **Pediatric** | | |
| --- | --- | --- | --- | --- | --- | --- | --- |
|  | P-One | P-PLUS | |  | P-ONE | P-PLUS | |
|  | CGTR | GTR2 | GTR1 |  | CGTR | GTR2 | GTR1 |
| Mean | 2.7 | 0.1 | 0.1 |  | 6.7 | 1.2 | 1.1 |
| SD | 1.8 | 0.1 | 0.3 |  | 2.4 | 1.0 | 0.9 |
| Max | 8.4 | 0.8 | 1.3 |  | 14.5 | 4.6 | 2.4 |
| Min | 0.0 | 0.0 | 0.0 |  | 2.3 | 0.0 | 0.0 |

**Table S5.** Average number of pediatric cases per day averaged across a given year for each treatment room. Underlined values represent years in which pencil beam scanning was utilized. Note that for GTR1, 2023 represents data averaged over half the year.

|  | **GTR3** | **GTR2** | **GTR1** | **CGTR** |  | **Total** |
| --- | --- | --- | --- | --- | --- | --- |
| 2018 | 1.8 | 3.1 | 15.2 | 0.0 |  | 20.1 |
| 2019 | 1.1 | 2.5 | 7.4 | 0.0 |  | 11.0 |
| 2020 | 0.8 | 0.7 | 0.0 | 5.0 |  | 6.5 |
| 2021 | 0.5 | 0.6 | 0.0 | 5.4 |  | 6.5 |
| 2022 | 0.1 | 1.5 | 0.0 | 6.7 |  | 8.3 |
| 2023 | 0.0 | 1.8 | 0.2 | 8.1 |  | 10.1 |
| 2024 | 0.0 | 1.3 | 1.8 | 8.8 |  | 11.9 |

**Table S6.** Percentage of prostate cases that had pelvic lymph node involvement and were treated with pencil beam scanning (PBS) in PBS capable rooms between 2018 - 2024. Note that for GTR1, 2023 represents data averaged over half the year.

|  | **2018** | **2019** | **2020** | **2021** | **2022** | **2023** | **2024** |
| --- | --- | --- | --- | --- | --- | --- | --- |
| GTR2 | 26% | 60% | 61% | 45% | 48% | 50% | 60% |
| CGTR |  |  | 0% | 32% | 46% | 39% | 57% |
| GTR1 |  |  |  |  |  | 33% | 33% |
| Total | 26% | 60% | 53% | 40% | 47% | 42% | 50% |

**Figure S1.** Polar histograms of gantry and couch angles used for brain treatments in Gantry Rooms 1 and 2 (GTR1 & GTR2) of the ProteusPLUS and Compact Gantry Room (CGTR) of the ProteusONE.

**Figure S2.** Polar histograms of gantry and couch angles used for prostate treatments in Gantry Rooms 1 and 2 (GTR1 & GTR2) of the ProteusPLUS and Compact Gantry Room (CGTR) of the ProteusONE.

**Figure S3.** Polar histograms of gantry and couch angles used for breast treatments in Gantry Rooms 2 (GTR2) of the ProteusPLUS.

**Figure S4.** Polar histograms of gantry and couch angles used for CNS treatments in the Gantry Rooms 2 (GTR2) of the ProteusPLUS and Compact Gantry Room (CGTR) of the ProteusONE.

**Figure S5.** Example patient with both shallow (ipsilateral pelvic nodes) and deep (prostate and contralateral pelvic nodes) target areas. Treating this patient in Gantry Room 2 (GTR2) of the ProteusPLUS requires the use of a 7.4 cm range shifter, which makes the maximum treatment depth 31.4 cm, subsequently requiring the maximum energy of the cyclotron. On the ProteusONE, this case can be treated without a ranger shifter and the maximum treatment depth remains 24 cm, requiring a cyclotron energy of only 195 MeV.
